# Primary open-angle glaucoma: everyone's business

**Published:** 2012

**Authors:** Hannah Faal

**Affiliations:** Chairperson: Africa Vision Research Institute, Durban, South Africa.

**Figure:**
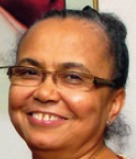
Hannah Faal

Primary open-angle glaucoma (POAG) is sometimes called the ‘thief of sight’. There is no pain or discomfort, and vision loss is so gradual that people often do not notice it.

POAG cannot be cured: it requires ongoing treatment for the remainder of a patient's life. Blindness from POAG also cannot be reversed, but it can be prevented if the disease is diagnosed early and treated.

Effectively addressing POAG therefore requires the careful involvement of many different people, including health workers, the patient, non-clinical staff, and health planners.

As **eye care practitioners**, we must do more than merely diagnose and treat people with POAG; we must gain the trust of patients and show them that we are there to help.

It is important to understand the fears people have about surgery, which is often the most certain way to preserve the sight of people with POAG. Many people dread the words ‘surgery’ or ‘operation’ but will also struggle to afford eye drops or come to the eye clinic for follow-up visits (see the case study on page 71). Unless we address their fears and misconceptions, they will certainly lose their sight.

**Patients** themselves, and their carers, are extremely important in the successful management of glaucoma, whether by surgery or medical treatment. They must come to the clinic for surgery and follow-up and instil any eye medication needed. Patients also can help to prevent blindness from POAG in their loved ones by encouraging their first-degree relatives (parents, siblings, and children) to come for an eye examination.

It can be very helpful to find out what the community knows and thinks about POAG (page 44). This knowledge enables us to provide appropriate information to patients and their families and ensures that we are effective in raising the public's awareness of the condition (page 46).

For patients on medical treatment, time is needed to explain the crucial role patients or their carers have to play, as well as all the practicalities (pages 77–79):

- what happens when they are not used
- how and when to instil eye drops
- where to get eye drops, where to keep them, and how much they cost
- how to recognise when their vision is deteriorating
- how to recognise fake drugs.

People who have been diagnosed with POAG will have a lot of new information to absorb, and they may struggle to come to terms with being told that they may lose their sight or – more frequently – with the fact that the sight they have already lost cannot be restored. Patients also have different ways of dealing emotionally with a diagnosis of POAG. They may postpone taking any action, go into denial, or may seek help from other providers, some of whom may have harmful practices.

We should do our best to understand how our patients feel and what they may be experiencing. We can use these insights to help them deal with the despair and despondency of having vision loss, for example by emphasising the residual vision which can be enhanced (with the help of low vision services and/or community-based rehabilitation) rather than the loss which cannot be retrieved. Strong links with low vision services and community-based rehabilitation is therefore an essential component of glaucoma care.

**Figure:**
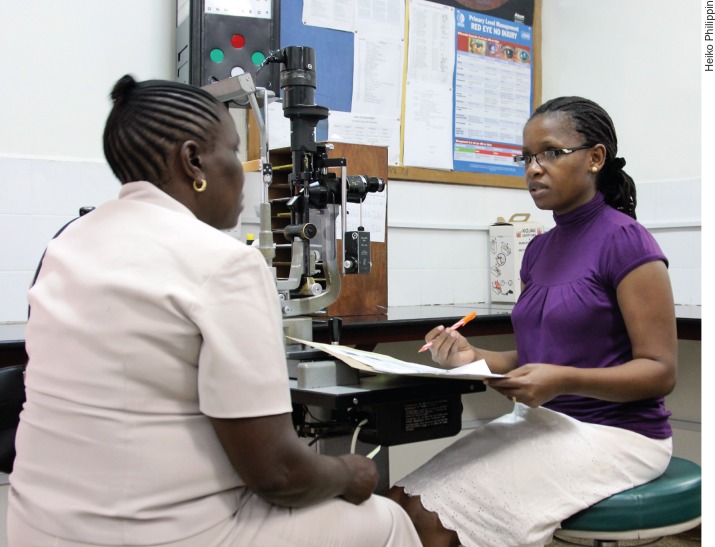
Patients with open-angle glaucoma need our support in order to understand how they can help prevent further sight loss from this life-long disease. TANZANIA

POAG is very similar to other non-communicable diseases, such as high blood pressure and diabetes, where the disease causes damage before there are any signs or symptoms to observe. As with these conditions, regular measurements have to be made, patients must adhere to advice about the use of medications. Close relatives also may be at risk and have to be examined. We may be able to learn a lot from our colleagues about how they manage these conditions and how we can apply their experience to the management of POAG. For example, patients who are struggling to accept a diagnosis of POAG would find tapping into the strengths of a POAG patient support group to be of great help.

We must give our patients enough of our time, and if our own time is limited, then we should arrange for them to speak with a trained counsellor. This will be someone who understands the condition and the treatments available but who also knows how to listen to patients and help them make the best decisions for their eye health and future vision.

**Facts about primary open-angle glaucoma**

- Worldwide, 45 million people were estimated to have POAG in 2010
- A total of 4.5 million (10%) were estimated to be blind as a result
- Relative to population size, there are up to four times more cases of POAG in people of African origin than in other ethnic groups.

*Source*: Johnson GJ et al. The epidemiology of eye disease (3rd edition). London, UK: Imperial College Press (13 May 2012).

Although the medical and surgical treatment of POAG is the responsibility of ophthalmic staff at the tertiary or secondary level, **people working in primary health care** have a major role to play in the counselling of patients and their relatives who are also at risk. After diagnosis, they can help to provide support to patients regarding low vision services and the use of medications. **Mid-level personnel** with good training and supervision can help by taking regular measurements (intra-ocular pressure and visual fields), capturing and transferring information and images, and making timely referral of patients according to agreed clinical guidelines and protocols.

**Optometrists** have a special role to play: routine checks for POAG within the presbyopia age group will help with early diagnosis and referral. They could provide regular monitoring of people who are at risk of developing POAG.

**Non-clinical eye care staff** also have a very important role to play in glaucoma care and it is important that they are well informed (see panel below).

- **Receptionists** are the first contact for people with POAG. They may have more time to talk with patients. They also speak the same kind of ‘non-medical’ language as patients, and may therefore have a greater influence over the opinions of patients. If they are informed, less harm is done.
- **Records staff** must understand why it is so important to have well-kept and accurate records, as these will help to reveal the trend in the progression of POAG.
- **Technicians** are responsible for maintaining and servicing eye care equipment essential to the provision of glaucoma care. Poorly calibrated or non-functioning tonometers can result in inaccurate test results, which means that patients will receive the wrong care. Tonometers must be calibrated regularly and every user must know how to do this (page 65). A surgical instrument set that is not complete or well maintained can also contribute to poor surgical outcomes. We should encourage entrepreneurial attempts to make technology affordable, including image capture and transfer for diagnosis as well as laser treatment. Eventually, there will be technology to help with early diagnosis of glaucoma, which will also allow patients to self-diagnose, keep accurate records, and track disease progression.
- We need **pharmacists** who are willing to stock the right glaucoma drugs and know where to procure them. If pharmacists have comprehensive knowledge of what impact glaucoma has on patients, families, and society, they can advocate for and prioritise glaucoma medications for inclusion in essential drugs packages.

Fear of job loss and the perceived stigma of vision loss contributes to the secrecy of some glaucoma patients. We must engage with **employers** and encourage them to help to reduce the stress for people with glaucoma. They can do so by providing insurance coverage for health checks and treatment, and by offering some assurance of continued employment (even if a change of roles is necessary to suit the person's reduced vision).

**Health planners** may find that a strategic approach similar to that used for non-communicable diseases such as diabetes and hypertension may have benefits. This might include conducting surveys, making drugs affordable, and advocating for the inclusion of glaucoma in policies relating to non-communicable diseases and universal coverage.

When POAG is truly made everyone's business, it will be possible to achieve control and reduce its devastating impact.

**What non-clinical staff should know**

**Figure:**
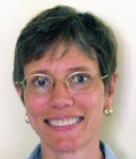
**Susan Lewallen** Co-director, Kilimanjaro Centre for Community Ophthalmology, www.kcco.net

Understanding a few simple facts about POAG will help non-clinical members of the eye care team to work in tune with the clinical staff and to understand what patients are facing.

In particular, it is useful for them to understand how POAG differs from cataract (page 62).

- There are different types of glaucoma so diagnosis and treatment is more difficult than for cataract.
- People with POAG are often unaware that they have the condition until significant vision has been lost.
- Visual loss from glaucoma cannot be reversed; treatment aims to stop or slow the visual loss.
- Because vision lost from POAG cannot be restored, early detection is critical.
- Accurate diagnosis of POAG in the early stages can be difficult, even for highly-trained ophthalmologists.
- Treatment may involve surgery or eye drops.
- Eye drops, if used, will have to be used regularly and for the remainder of the patient's life.
- Surgery will not restore vision; it stops or slows down the further loss of vision.
- By comparison to cataract surgery, patients need much closer follow-up after glaucoma surgery.
- ‘I??’ in the patient record refers to the pressure in the eye, or the ‘intra-ocular pressure’.
- IOP is important in diagnosis and management of glaucoma, as the aim of treatment is to lower the IOP.
- POAG is a chronic condition, like high blood pressure or diabetes. Patients will need care for the rest of their lives and keeping accurate records is vital.

**The patient's perspective**

**Aruna's story**

When I was first diagnosed it hit me hard. I thought – “Oh no, it cannot happen to me.” I was really quite distressed; I didn't know anything about glaucoma – all I knew was that I could go blind. I'm quite an independent person and I would hate to be dependent on anyone. The thought was very scary. I do have follow-up appointments at the hospital, but I think they should be more frequent than they are. Even at the eye clinic, the staff write down notes, but don't tell you the information. My daughter, who is a doctor, helped me to find out about glaucoma and the importance of keeping up with my treatment. Having the right information made me feel much better and gives me the independence to manage my condition well.

**It hit me hard. I was distressed; I didn't know anything’**

**John's story**

I was diagnosed with ocular hypertension, which then developed into glaucoma. The environment of the hospital and the way health professionals communicate with patients can affect our ability to live with the condition. Staff should be open, explain what is going on and how medicines work, so that it takes the worry out of the situation. One of the great difficulties with glaucoma is that I have no idea how it is progressing. I have to rely on what I am told, so I want to ask questions to understand. After all, if I wasn't worried I wouldn't ask! I visit two hospitals for visual field tests. In one, the test is done with no explanation while in the other the nurse will take time to go through the results. This is much better. It means I can see the difference for myself and can ask sensible questions of my ophthalmologist during my appointment.

*Reprinted with kind permission of the International Glaucoma Association*: www.glaucoma-association.com

